# Design, delivery, and determinants of uptake: findings from a food hygiene behavior change intervention in rural Bangladesh

**DOI:** 10.1186/s12889-022-13124-w

**Published:** 2022-05-04

**Authors:** Shafinaz Sobhan, Anna A. Müller-Hauser, Tarique Md. Nurul Huda, Jillian L. Waid, Om Prasad Gautam, Giorgia Gon, Amanda S. Wendt, Sabine Gabrysch

**Affiliations:** 1 Institute of Public Health, Charité – Universitätsmedizin Berlin, corporate member of Freie Universität Berlin and Humboldt-Universität zu Berlin, Charitéplatz 1, 10117 Berlin, Germany; 2 Research Department 2, Potsdam Institute for Climate Impact Research (PIK), Member of the Leibniz Association, Potsdam, Germany; 3 Heidelberg Institute of Global Health, Heidelberg University, Heidelberg, Germany; 4 International Centre for Diarrhoeal Disease Research, Bangladesh (icddr,b), Dhaka, Bangladesh; 5 WaterAid UK, London, UK; 6 Department of Infectious Disease Epidemiology, London School of Hygiene and Tropical Medicine, London, UK

**Keywords:** Child feeding, Behavior adoption, Implementation, Emotional driver

## Abstract

**Background:**

Microbial food contamination, although a known contributor to diarrheal disease and highly prevalent in low-income settings, has received relatively little attention in nutrition programs. Therefore, to address the critical pathway from food contamination to infection to child undernutrition, we adapted and integrated an innovative food hygiene intervention into a large-scale nutrition-sensitive agriculture trial in rural Bangladesh. In this article, we describe the intervention, analyze participation and uptake of the promoted food hygiene behaviors among intervention households, and examine the underlying determinants of behavior adoption.

**Methods:**

The food hygiene intervention employed emotional drivers, engaging group activities, and household visits to improve six feeding and food hygiene behaviors. The program centered on an ‘ideal family’ competition. Households’ attendance in each food hygiene session was documented. Uptake of promoted behaviors was assessed by project staff on seven ‘ideal family’ indicators using direct observations of practices and spot checks of household hygiene conditions during household visits. We used descriptive analysis and mixed-effect logistic regression to examine changes in household food hygiene practices and to identify determinants of uptake.

**Results:**

Participation in the food hygiene intervention was high with more than 75% attendance at each session. Hygiene behavior practices increased from pre-intervention with success varying by behavior. Safe storage and fresh preparation or reheating of leftover foods were frequently practiced, while handwashing and cleaning of utensils was practiced by fewer participants. In total, 496 of 1275 participating households (39%) adopted at least 5 of 7 selected practices in all three assessment rounds and were awarded ‘ideal family’ titles at the end of the intervention. Being an ‘ideal family’ winner was associated with high participation in intervention activities [adjusted odds ratio (AOR): 11.4, 95% CI: 5.2–24.9], highest household wealth [AOR: 2.3, 95% CI: 1.4–3.6] and secondary education of participating women [AOR: 2.2, 95% CI: 1.4–3.4].

**Conclusion:**

This intervention is an example of successful integration of a behavior change food hygiene component into an existing large-scale trial and achieved satisfactory coverage. Future analysis will show if the intervention was able to sustain improved behaviors over time and decrease food contamination and infection.

**Supplementary Information:**

The online version contains supplementary material available at 10.1186/s12889-022-13124-w.

## Background

An estimated 149 million children under 5 years of age worldwide suffer from chronic undernutrition [1]. Particularly during the first 1000 days of life, undernutrition can have detrimental developmental consequences – including impaired cognitive development, compromised immune function, and increased risk of disease – and prevent children from reaching their full potential and productivity in adulthood [2].

Key causes of undernutrition in children include insufficient intake of nutritious food as well as poor sanitation and inadequate food hygiene practices – leading to repeated enteric infections and reduced nutrient uptake in the gut [3]. Most interventions addressing child undernutrition target the pathway of nutrient intake, ensuring that the child receives the right amount of nutritious food at the right frequency. Microbial contamination of food has received comparatively little attention in nutrition programs, although it is a known contributor to diarrheal disease and highly prevalent in low-and middle-income settings [4, 5]. From 6 months of age, it is important to complement breast milk with other foods to achieve adequate nutrition. However, unhygienic preparation and feeding frequently expose children to microbially contaminated complementary food, thus putting them at risk of ingesting pathogenic bacteria and developing intestinal infections and diarrheal disease [4–7].

Consistent adoption of handwashing and food hygiene practices can considerably reduce microbial food contamination and thereby diarrheal incidence [8–10], however, in many settings, consistent practice of these behaviors remains challenging [10]. In Bangladesh, research shows that although knowledge about handwashing is widespread, handwashing at certain critical time points (e.g. before cooking and serving food) is rarely practiced [11] and not easily improved by large-scale WASH programs [12].

Changing behaviors, especially habitual ones, is challenging. Behavior is determined by various factors, like the physical environment [13, 14], social norms, and own beliefs and habits. Therefore, to facilitate behavior change, interventions should address multiple determinants of behavior [15]. Recent studies showed that behavior change can be successfully induced by vigorously advocating and frequently promoting essential food hygiene practices as well as using emotional drivers [14, 16–27]. For instance, a study in Nepal conducted by Gautam and colleagues used emotional drivers (such as nurture, status, affiliation and disgust) as well as attractive and engaging group activities (including games and competitions) and repeated individual household visits to improve food hygiene practice [14, 26]. Physical change in kitchen settings was also encouraged to reinforce and facilitate the targeted new behaviors (e.g. hand-washing station with soap close-by, eye-danglers as reminders) [14, 26]. During pilot studies, this behavioral approach resulted in a significant improvement in food hygiene behaviors and reduction in bacterial food contamination [14, 24–26]. However, such an approach has not yet been used in any larger studies, nor examined over a longer time period. Inspired by the Nepali trial, we adapted and scaled up their food hygiene intervention package and training modules, integrating this into a large-scale nutrition-sensitive agriculture trial in rural Bangladesh to address the critical pathway from food contamination via infection to malnutrition [28].

After describing the design and implementation of this innovative food hygiene behavior change intervention in Bangladesh, we aim to (1) assess the level of participation in food hygiene sessions and the uptake of promoted behaviors among participating households during implementation and (2) identify the underlying determinants that facilitated the adoption of food hygiene behaviors among the target population.

## Methods

### Study setting and population

**Fig. 1:**
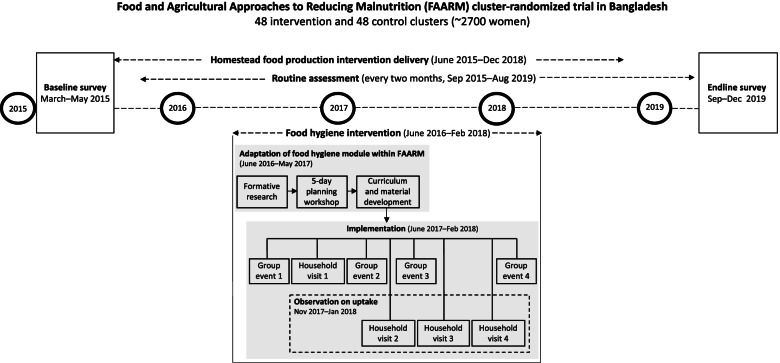
Design and implementation of the food hygiene intervention within the FAARM trial in Bangladesh

The study is set within a homestead food production program implemented by Helen Keller International in two rural sub-districts in Habiganj, Bangladesh, as part of the “Food and Agricultural Approaches to Reducing Malnutrition” (FAARM) cluster-randomized trial (2015–2019). FAARM included 2700 young married women in 96 settlements (geographic clusters): 48 intervention and 48 control. Participating women in intervention settlements received trainings on year-round gardening, poultry rearing, nutrition and hygiene from mid 2015 to late 2018 [28]. While achieving diversified production and improved nutrition practices was a priority in Helen Keller International’s homestead food production training curriculum, activities to improve food hygiene were limited to messages on handwashing and instructions on constructing handwashing stations. To promote hygiene behaviors around food preparation and child feeding more intensively, an additional behavior change component was designed and delivered to 1275 intervention women in all 48 intervention clusters over 8 months from July 2017 to February 2018. We collected data on these women during intervention delivery to understand participation and uptake. A comparison to control settlements was outside the scope of the present analysis. Figure 1 shows the detailed design and implementation of the food hygiene intervention in the FAARM trial.

### Design of the food hygiene intervention

The design and development of the food hygiene intervention, adapted from the Nepali model to the FAARM setting and population, were undertaken in three steps:

The first step involved formative research, using interviews with 423 FAARM participant women and semi-structured observations in 36 households, to learn about their environmental conditions, their existing food preparation, food storage, child feeding, and hygiene practices. Five focus group discussions with 6–10 participants each, including a motive mapping exercise, were also done to understand women’s psychological motives that could potentially influence their current food hygiene behaviors.

In the second phase, a five-day planning workshop – run by the creator of the Nepali food hygiene intervention and attended by FAARM researchers, project technical officers, and field facilitators – introduced in detail the Nepali food hygiene curriculum, materials, and delivery model. Additionally, the team assessed FAARM’s context, which guided the adaption process to maximize local acceptance and cultural appropriateness.

In the third step, the FAARM implementation team synthesized the findings from the earlier two steps and altered aspects of the Nepali intervention to ensure a good fit between FAARM’s on-going activities and needs of the target population while maintaining the theoretical framework of the original model. Two major modifications were the integration of optimal feeding and eating behaviors for children and women, and changes in program delivery in terms of scale, intensity, and duration of the intervention. In FAARM, the food hygiene intervention was delivered at 10 times the scale: to 1275 women in 48 settlements compared to 120 women in 4 settlements in Nepal. To maintain feasibility and balance with other FAARM activities, we conducted eight sessions over 8 months compared to 12 sessions over 3 months in Nepal. Table 1 provides an overview of the adaptation of the Nepali food hygiene model to the FAARM context.

**Table 1:** Overview of key changes in adapting the Nepali food hygiene promotion model to the FAARM context in Bangladesh

	Nepal	Bangladesh	Key changes and rationale
**Objective**	Developing an innovative food hygiene intervention using a behavior-centered design approach in Nepal	Adapting and integrating the Nepali innovative food hygiene package within a large-scale nutrition-sensitive intervention in Bangladesh	FAARM combined food hygiene with ongoing agricultural and nutrition support to address the critical pathway from food contamination via infection to undernutrition
**Contents and focus**			
Targeted food hygiene behaviors	**5 food hygiene behaviors**	**6 optimal feeding and food hygiene behaviors**	Two optimal feeding/eating related behaviors for children and mothers were added to reinforce ongoing nutrition messages of FAARM. The microbial contamination of tube well water (which is the primary drinking water source in rural Bangladesh) is relatively low at source; and the risk of contamination increases during household water storage and handling [29, 30]. Boiling would also be a huge effort. Therefore, the message on boiling water and milk was taken out and emphasis was put on safe storage of drinking water at the household level.
	• Handwashing with soap • Cleanliness of serving utensils • Safe storage of cooked food • Thorough reheating • Boiling of milk and water	• Exclusive breastfeeding • Dietary diversity for women and children • Handwashing with soap • Cleanliness of serving utensils • Safe storage of cooked food and drinking water • Cooking fresh or thorough reheating	
Motivational drivers	Nurture	i **‘Nurture’** or boosting caregivers’ desire for their children’s optimal health and wellbeing ii **‘Disgust’**, i.e., triggering strong negative feelings toward activities or habits that are associated with the risk of infections and diseases by highlighting the links between poor hygiene practices and transmission of germs iii **‘Affiliation and pride’**, i.e., creating a feeling of social togetherness and achievement derived from making healthier choices and being an inspiring figure for others	FAARM developed a promotional strategy that was built around a similar set of emotional drivers; however, the triggers of these drivers were adapted culturally.
	Disgust		
	Affiliation		
	Social respect/status		
Central theme	***Ideal mother - safe food, healthy child. ***This theme portrayed a central ‘ideal mother’ character, who practiced safe hygiene to be respected in the community	***Safe and nutritious food: ideal family. ***This theme communicated the idea that giving a child a nutritious and diverse diet and handling foods safely will help the family to enjoy a healthy and happy life and earn a sense of pride in the community	The focus was shifted from mother to family, recognizing the fact that family members play a powerful role in influencing each other’s behaviors and that a mother’s ability to adopt a healthy behavior strongly depends on family support in our context.
**Scale and intensity**			
Setting	**4 intervention settlements** with 30 households per settlement	**48 intervention settlements** of the FAARM trial with 10 to 65 eligible women per settlement	In FAARM, the intervention was delivered at a more than 10 times larger scale. To maintain feasibility and balance considering other FAARM activities, it was designed to be implemented over a longer time frame at a lower intensity. While the Nepali intervention was only targeted to women with young children, FAARM targeted all women in the intervention arm of the trial.
Study participants	**Primary target group:** Mothers with a child aged 6–59 months **Secondary target groups:** Grandmothers, community people and school students **Total targeted:** 120 women and their household members	**Primary target group:** Married women less than 30 years old at enrollment **Secondary target groups:** Husbands, mothers-in-law, other family members **Total targeted: **1275 women and their household members	
Duration	**3 months**	**8 months**	
Frequency of contract	Every 15 days; a joint community/group event was followed by a door-to-door household visit	Once every month; a group event was usually followed by a household visit	
Dissemination channels	**12 structured sessions conducted by 15 food hygiene motivators** • 2 community events • 4 group events • 6 household visits **Other touch point: **Half-day school sessions with students and teachers in four government schools Ideal mother’s photos put-up in the junction of the village for social respect and pride	**8 structured sessions conducted by 8 food hygiene promoters (FHPs)** • 4 group events • 4 household visits	Community touch points were removed from the implementation design to reduce spillover to FAARM control settlements.

Once the key behaviors and messages were finalized, the implementation guideline was adapted (e.g., changes in text, names, storylines, etc.) to accommodate added behaviors and messages and later translated into Bengali. A professional graphic artist helped to redesign all illustrations and communication materials to reflect local context. Afterwards, all prototypes were pretested in a small group of households with similar demographic backgrounds as the FAARM participants, and changes were made based on their feedback.

To implement food hygiene activities, eight female Food Hygiene Promoters (FHP) were hired from the local area. Before rolling out the activities, the FHPs received a five-day training on the implementation of the curriculum and materials. In addition, they attended a one-day refresher training every month to exchange lessons learned, review their progress, receive materials, and plan for the next activity.

### Content of the food hygiene intervention

The FAARM food hygiene intervention used a behavior-centered approach to promote six key optimal feeding and food hygiene behaviors (Table 1) among participating households by encouraging changes to their physical settings and using emotional drivers such as nurture, disgust, affiliation, and pride. The intervention was rolled out through eight structured sessions: four group events and four household visits (Table 2).

**Table 2:** Structure of the food hygiene intervention

Session	Purpose	Activity	Behavior change technique^a^
Group event 1	Introduce 6 key feeding and food hygiene behaviors and present their importance for the health and well-being of the child	- Storytelling	- Information about health consequences
		- Picture matching game	- Instructions on how to perform the behavior
	Encourage households to grow a diverse garden and maintain a clean kitchen and homestead compound along with the 6 key behaviors	- Women’s pledge to adopt the key behaviors and ‘ideal family’ practices - Announcement of ‘ideal family’ competition and indicators	- Goal setting (behaviors) - Commitment - Social incentive
Household visit 1	Physical rearrangement of kitchen or cooking area as a cue to perform the new behaviors	- Kitchen/cooking area makeover - Providing reminder materials (e.g., eye danglers, stickers) for kitchen	- Restructuring the physical environment - Adding objects to the environment
	Highlight the links between cleanliness of kitchen and 4 food hygiene behaviors	- Introduction to ‘clean kitchen’ indicators	- Instructions on how to perform the behavior - Goal setting (behaviors)
Group event 2	Remind of the consequences of poor feeding and food hygiene behaviors for young children	- Sharing individual experiences with the group	- Review behavior goal(s)
		- Role play: child life game	- Instructions on how to perform the behavior
		- Recall of food hygiene key messages - Announcement of ‘clean kitchen’ competition	- Behavior practice/rehearsal - Social incentive
Household visit 2	Highlight the importance of diversified, nutritious, and safe food for women of child-bearing age and young children	- Discuss individual household experiences	- Review behavior goal(s)
		- Discuss the importance of a garden with vegetables and fruit in improving household’s dietary diversity	- Information about health consequences
		- Flip chart presentation on optimal feeding behaviors	- Instructions on how to perform the behavior
		- Demonstration of the hygienic preparation of a diverse food plate for women and young children (6–23 months) - Distribution of a complementary feeding mat	- Demonstration of the behavior - Adding objects to the environment
		- Observation of food hygiene activities against ‘ideal family’ and ‘clean kitchen’ indicators	- Feedback on behavior
Group event 3	Demonstrate transmission of germs and food contamination to emphasize the importance of clean utensils and handwashing	- Sharing individual experiences with the group	- Review behavior goal(s) - Feedback on behavior
		- A ‘disgust exercise’ using Glo Germ™. Glo Germ™ is a fluorescent liquid or gel visible only under ultraviolet light. It was used to simulate the distribution of germs on hands and utensils during food hygiene sessions.	- Information about health consequences - Instructions on how to perform the behavior
Household visit 3	Highlight the importance of proper storage of food and drinking water and thorough reheating of stored food	- Discuss individual household experiences - Flip chart presentation on food hygiene behaviors	- Review behavior goal(s) - Information about health consequences
			- Instructions on how to perform the behavior
		- Demonstration of proper time and temperature of safe proper reheating using food thermometer	- Demonstration of the behavior
		- Observation of food hygiene activities against ‘ideal family’ and ‘clean kitchen’ indicators	- Feedback on behavior
Household visit 4	Ask women to self-assess their food hygiene practices	- A ‘pile sorting exercise’ to rank the behaviors in order of ease to practice	- Graded tasks
	Encourage maintenance of practices in future	- Observation of food hygiene activities against ‘ideal family’ and ‘clean kitchen’ indicators	- Feedback on behavior
Group event 4	Announce the ‘ideal family’ and the ‘clean kitchen’ winners	- Reward ceremony and distribution of ‘ideal family’ photos among ‘ideal family’ winners and handwashing soaps among ‘clean kitchen’ winners	- Social reward
		- Selection of peer leaders	- Social support
		- Public pledge to continue food hygiene practices	- Commitment

^a^Behavior change techniques taxonomy (v1) [31] was used to label the intervention activities

A group event was a one-hour participatory courtyard session with a group of 5–25 women. These food hygiene sessions were also open to other family members, especially husbands and mothers-in-law. Every group event commenced with a series of routine activities such as i) welcoming participants with a jingle conveying key food hygiene messages; ii) setting up a handwashing station with soap at a corner of the venue to encourage the participants to wash hands before taking a seat; iii) wearing a badge showing the ‘ideal family’ logo. Every group event then focused on a different topic, using fun materials like a hand fan invitation card, germ simulation experiment with Glo Germ™ liquid and ultraviolet light, etc. and facilitated participatory discussions and playful engagement of participants through storytelling, role play, and simulation games to communicate key messages and highlight benefits of practicing key behaviors at home.

**Fig. 2:**
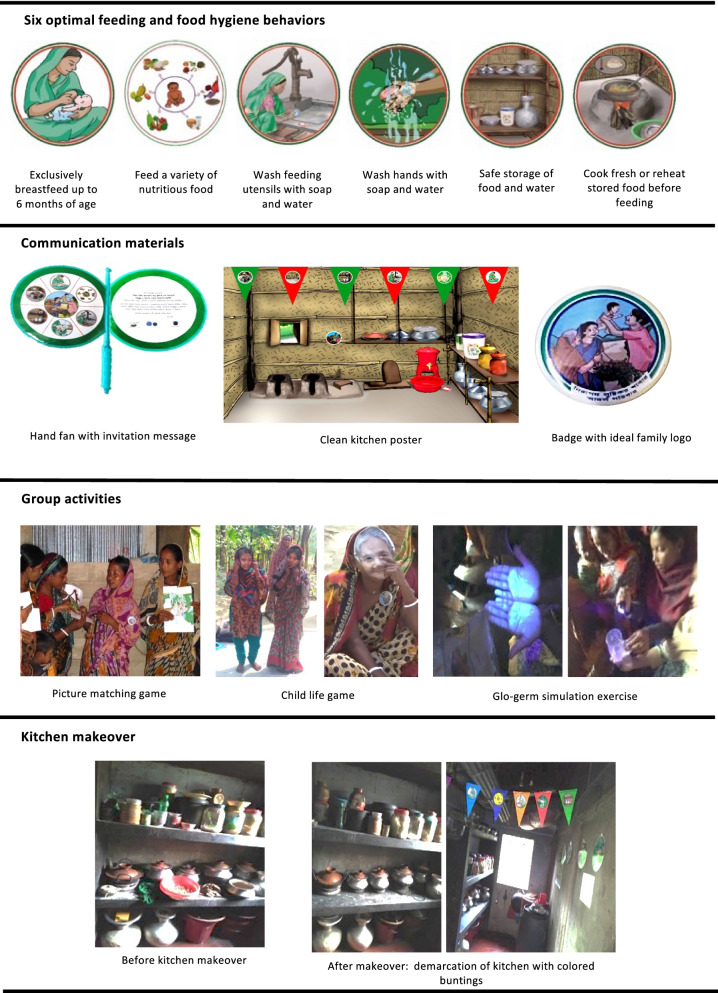
Pictures of communication materials and key food hygiene activities

In addition to the four group events, the FHPs conducted four visits to each woman’s household. These household visits were designed to help families change their physical settings, including demarcation of the cooking area with colored flags and buntings of promoted behaviors, demonstration of ideal food hygiene practices, installation of a handwashing station, placing of reminder stickers with the six key behaviors in locations that were visible to family members to act as visual cues to practice appropriate behaviors. In addition, families received practical support during FHP visits, such as demonstration of a diverse food plate for mother and child or use of a food thermometer to demonstrate temperature and time for proper reheating of leftover food. During visits, FHPs also offered support to solve individual challenges in order to increase each household’s capability and adoption of safe food hygiene behaviors. Figure 2 presents pictures of some materials and key activities.

Important highlights of the food hygiene intervention were the ‘ideal family’ and ‘clean kitchen’ competitions as drivers for optimal feeding and safe food hygiene practices. We developed two sets of indicators (Table 3) reflecting promoted behaviors to determine the winners of the competitions who received a small reward at the end of the intervention. In addition, each time the women participated in a session, they received a small gift (such as soap, dish washing powder, a feeding mat, etc.) as an encouragement to attend the session and a support for improving their food hygiene practices at home. To promote sustained behavior adoption, after the end of the intervention, each group selected one or two peer leaders among the winners of the ‘ideal family’ and ‘clean kitchen’ awards, motivated to support their respective group members to continue the practices in the future.

**Table 3:** Indicators for the competitions on ‘ideal family’ and ‘clean kitchen’

**‘Ideal family’ indicators**	
	Having a garden with diverse vegetables and fruits, i.e., at least two types of green leafy vegetables, two types of other vegetables, and one fruit tree
2.	Woman and children are eating a variety of nutritious foods: besides rice, the daily menu includes green leafy vegetables, other vegetables, fish/meat/liver/egg, thick lentils, and seasonal fruits
3.	Washing utensils with soap and clean water before preparing and serving food
4.	Washing hands with soap and clean water before preparing food, feeding a child, and/or eating
5.	Storing foods and drinking water fully covered and above the ground
6.	Fresh cooking/reheating food thoroughly each time before feeding /eating
7.	Keeping the kitchen and homestead compound clean and free from animal/chicken feces and other rubbish
**‘Clean kitchen’ indicators**	
1.	Clean and demarcated kitchen
2.	Hand-washing station (with soap and water) inside the kitchen or next to the kitchen
3.	Rubbish kept in a covered container/place and emptied regularly so it does not attract flies
4.	Separate area for poultry and other animals if these are kept inside

### Data sources

Three different data sources were used for analysis: (i) food hygiene administrative data including participation lists, structured observations and household spot checks (used for the competitions), (ii) the FAARM 2015 baseline survey and (iii) data from selected rounds of the routine assessment component of the FAARM surveillance system which interviewed all trial participants every 2 months from 2015 to 2019 to assess impact pathway indicators [28].

Data for this study were primarily gathered through three rounds of direct observation, carried out during household visit 2 (November 2017), household visit 3 (December 2017), and household visit 4 (January 2018). The FHPs used a short, structured checklist to collect information on women’s current practice relating to the ‘ideal family ‘and ‘clean kitchen’ indicators. They also conducted spot checks to collect household environment data, which included the presence of a garden for homestead food production, cleanliness of the kitchen and household environment, availability of a handwashing device and safe food storage facilities. The FHPs performed the direct observation and the spot checks silently during a household visit that lasted approximately an hour. They also collected dietary diversity data through a 24 hour recall, in which a woman was asked to report all the foods and beverages consumed by her and her young child in the past 24 hours. Each household’s participation at group events and household visits was compiled from field registers.

Data related to household characteristics (e.g., household wealth, structure) and women’s characteristics (e.g. women education, empowerment) were taken from the FAARM baseline survey conducted in 2015 [28]. Wealth quintiles were calculated using Principal Components Analysis, adapted from methods used by the Bangladesh Demographic and Health Survey [32]. Women’s education was assessed as the number of school years completed. As defined in a previous study on the FAARM population [33], women’s empowerment was operationalized as a woman’s ability in four domains: participation in intra-household decision-making, mobility outside the homestead compound, social support, and communication with husband and other women about issues such as health and education. Based on survey responses, women were categorized on their ability to exercise empowerment in each area on a scale with 3–4 categories ranging from unable to able [33]. Later, classification was further summarized into an empowerment variable, categorized as no or very little empowerment, some empowerment, greater empowerment.

Data on the number of children for each woman and the age of the youngest child were derived from routine assessment round 11 (May–June 2017), which was right before the beginning of the food hygiene intervention in July 2017. Data collected through interview questions in a sub-population of FAARM households during routine assessment round 8 (November–December 2016) were used as a pre-intervention reference for four food hygiene behaviors and dietary diversity. Similarly, round 10 (March–April 2017) and round 11 (May–June 2017) served as pre-intervention references for diverse garden practice. The different data sources and collection periods for the variables used in this article are summarized in Supplementary Table 1 in Additional file 1. All data for FAARM baseline survey and routine assessment rounds were collected with tablets using Open Data Kit software [34].

### Variables

For the analytic component of the study, we considered households’ participation in eight food hygiene sessions as the main exposure of interest. A household was considered to have participated in a session if either the woman herself or another adult household member was able to attend a group session or was present during a household visit. The level of participation was divided into three groups to define households with low (0–4 sessions), medium (5 or 6 sessions), or high (7 or 8 sessions) participation.

The two primary outcomes of the study were being an ‘ideal family’ or ‘clean kitchen’ competition winner, measured by selected indicators that reflected uptake and practice of promoted behaviors among intervention households. The ‘ideal family’ characteristics included 7 indicators (Table 3). Direct observation was done during household visits and the FHPs coded each indicator as ‘positive’ to denote that the activity was performed correctly and ‘negative’ to indicate otherwise. An ‘ideal family’ title was awarded if a household scored positive for at least 5 of the 7 indicators in each of the three observation rounds. Similarly, a ‘clean kitchen’ title was awarded if a household maintained at least 3 of the 4 promoted ‘clean kitchen’ activities, (Table 3) in each of the three assessments.

We selected household or woman characteristics as covariates if they could influence both participation in the food hygiene intervention and the practice of the optimal feeding and food hygiene behaviors. At the household level, we included household wealth, religion, number of household members, number of rooms in the house, size of homestead and agricultural land in our statistical analyses. As women’s characteristics, we considered education, empowerment, the number of children under 3 years of age and the age of the youngest child at the beginning of the food hygiene intervention.

### Statistical analysis

We described exposure and outcome variables, as well as further household and women’s characteristics using proportions for categorical and means and standard deviations for continuous variables. We used mixed effect logistic regression to examine the determinants of practicing food hygiene behaviors in study households, using settlement-level random effects. Data processing and analysis were carried out using Stata IC version 14.2.

## Results

### Sample characteristics

An overview of household and women’s characteristics for the 1275 women in the intervention arm are presented in Table 4. Seventy-one percent of households in our study population were Muslim, with the remainder Hindu, and households had on average 7 members. Most women had at least some education, while 16% never went to school. At the beginning of the food hygiene intervention, almost half of the women had at least one child under 3 years of age, 5% had two children in this age range.

**Table 4:** Characteristics of intervention households in Habiganj District, Sylhet Division, Bangladesh

Household characteristics		freq.	%
**Wealth**	Poorest	283	22.4
	Lower	255	20.2
	Medium	252	20.0
	Upper	257	20.3
	Wealthiest	217	17.1
**Religion**	Muslim	898	70.7
	Hindu	373	29.3
**Household members**	Up to 5	446	35.3
	5–10	585	46.3
	More than 10	233	18.4
**Women’s education**	None	198	15.6
	Partial/complete primary	562	44.2
	Partial secondary or more	511	40.2
**Number of children under 3 years**	No child	685	53.7
	One child	522	41.0
	Two children	68	5.3
**Age of youngest child** (under 3 years)	0–6 months	83	14.1
	7–12 months	125	21.2
	13–24 months	190	32.2
	25–36 months	192	32.5

Total *n* = 1275, for some variables total n is smaller due to additional missing values: wealth and number of household members (*n* = 1264), religion and women’s education (*n* = 1271)

### Participation in the food hygiene intervention

More than three quarters of households showed a high level of participation, with attendance in at least 7 out of 8 food hygiene sessions, while 8% of households only participated in 4 or fewer sessions (Table 5). Participation in household visits was slightly greater (on average 90%) than in group events (around 84%) (Additional file 2). A total of 1022 (80%) women participated in all three household visits which served as observation rounds to assess specific behaviors. Analyses concerning uptake of behaviors were performed in this subset.

**Table 5:** Participation intensity, ‘ideal family’ and ‘clean kitchen’ winners of the food hygiene intervention

		freq.	%
**Participation**	Low (0–4 sessions)	104	8.2
	Medium (5–6 sessions)	195	15.3
	High (7–8 sessions)	976	76.5
**‘Ideal family’ winner**^a^		496	38.9
**‘Clean kitchen’ winner**^b^		649	50.9

Total *n* = 1275

^a^ ‘Ideal family’ winner: household scored positive on at least 5 of 7 ‘ideal family’ indicators

^b^ ‘Clean kitchen’ winner: household scored positive on at least 3 of 4 ‘clean kitchen’ indicators

(See Supplementary Table 2, Additional file 2 for detailed participation in each session)

### Uptake of key optimal feeding and food hygiene behaviors

**Fig. 3:**
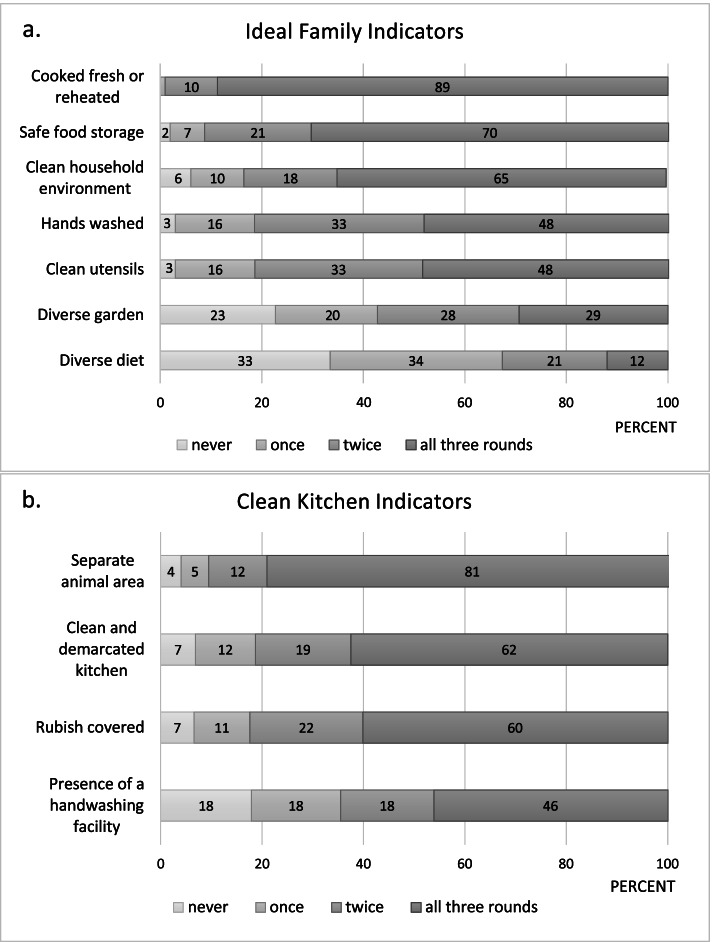
Practice of key behaviors composing the ‘ideal family’ and ‘clean kitchen’ indicators. **a** ‘Ideal family’ indicators. **b** ‘Clean kitchen’ indicators. Practice of ‘ideal family’ and ‘clean kitchen’ behaviors (in % of households) were assessed over three observation rounds, ranging from never practiced (lightest grey) to always practiced (darkest grey). This graph only shows households that could be observed for ‘ideal family’ and ‘clean kitchen’ indicators during all three observation rounds (*n* = 1022), households with less than 3 observation rounds were excluded (missing values: 253)

The specific hygiene behaviors were taken up and practiced with varying success. Safe storage was observed in 70% and fresh cooking or reheating of leftover foods in 89% of households in all three observation rounds, while handwashing before food preparation and child feeding, and cleaning of utensils were consistently practiced in only about half of households (Fig. 3a). Uptake of hygiene-related behaviors substantially increased from the levels seen before the food hygiene intervention. However, for some behaviors, the percentage of households practicing these declined slightly over time (See Supplementary Fig. 1, Additional file 3). Although the practice of nutrition-related behaviors (i.e., the consumption of a diverse and nutritious diet for women and children, and the presence of a garden with a variety of vegetables and fruits) was generally low in the study population (Fig. 3a), these showed a steady increase in practice throughout the observation period (Supplementary Fig. 1, Additional file 3).

Uptake of ‘clean kitchen’ practices was also mixed. Separation of animals from the kitchen was the most frequently observed ‘clean kitchen’ practice. In contrast, a functioning handwashing facility in or near the kitchen area was present in less than half of study households in all three observation rounds (Fig. 3b), in line with the poor handwashing and utensil cleaning practices of many households.

Based on their practice of all promoted behaviors, at the end of the intervention, 496 (39%) families were classified as an ‘ideal family’, and 649 (51%) households were classified as ‘clean kitchen’ winners (Table 5).

### Determinants of ‘ideal family’ and ‘clean kitchen’ winners

An increased level of participation in intervention activities was strongly associated with being considered an ‘ideal family’ or a ‘clean kitchen’ winner. Households that participated in at least 7 of 8 food hygiene sessions were much more likely to adopt promoted ‘ideal family’ and ‘clean kitchen’ practices and become a winner (for ‘ideal family’ OR: 9.5, and for ‘clean kitchen’ OR: 18.1) compared to households that participated in 4 or fewer sessions (See Supplementary Table 3, Additional file 4). Also, a range of household and women’s characteristics showed an association with being classified as an ‘ideal family’ or ‘clean kitchen’ winner in bivariable models (See Supplementary Table 3 and 4, Additional file 4).

In multivariable models, we found that both outcome measures (‘ideal family’ and ‘clean kitchen’ award) were strongly associated with household participation, household wealth, maternal education, and religion (Table 6). The odds of being an ‘ideal family’ or ‘clean kitchen’ winner were higher among households with high participation in the food hygiene intervention (for ‘ideal family’ AOR: 11.4 and for ‘clean kitchen’ AOR: 26.5); among Hindu households (for ‘ideal family’ AOR: 1.8 and for ‘clean kitchen’ AOR: 2.4); and for women who had at least some secondary education (for ‘ideal family’ AOR: 2.2 and for ‘clean kitchen’ AOR: 2.1). Poorer households were less likely to classify as an ‘ideal family’ (AOR: 0.4) or ‘clean kitchen’ winner (AOR: 0.3), while the richest households were more likely to win the ‘ideal family’ (AOR: 2.3) or ‘clean kitchen’ competition (AOR: 2.6), compared to households with intermediate wealth. Greater women’s empowerment was associated with being an ‘ideal family’ (AOR: 1.6) or ‘clean kitchen’ (AOR: 1.6) winner. Interestingly, the size of agricultural land was inversely related to winning the ‘ideal family’ or ‘clean kitchen’ competition (Table 6), in spite of the production component of being an ‘ideal family’.

**Table 6:** Adjusted associations of household and women characteristics with classification as ‘ideal family’ or ‘clean kitchen’ winner

		‘Ideal family’			‘Clean kitchen’		
Characteristics		OR	95% CI	***p***-value	OR	95% CI	***p***-value
**Participation**	Low	Ref.			Ref.		
	Medium	4.6	1.9–10.5	< 0.001	8.6	3.8–19.5	< 0.001
	High	11.4	5.2–24.9	< 0.001	26.5	12.3–57.0	< 0.001
**Wealth ***(in quintiles)*	Poorest	0.4	0.2–0.6	< 0.001	0.3	0.2–0.5	< 0.001
	Lower	0.9	0.6–1.3	0.5	0.6	0.4–0.95	0.02
	Medium	Ref.			Ref.		
	Upper	1.8	1.2–2.7	0.006	1.3	0.8–2.0	0.3
	Wealthiest	2.3	1.4–3.6	< 0.001	2.6	1.5–4.5	< 0.001
**Religion**	Muslim	Ref.			Ref.		
	Hindu	1.8	1.3–2.5	< 0.001	2.4	1.6–3.5	< 0.001
**Household members**	Up to 5	Ref.			Ref.		
	6–10	0.7	0.5–0.9	0.02	0.6	0.4–0.8	0.002
	More than 10	1.0	0.6–1.5	0.9	0.6	0.4–1.02	0.06
**Number of rooms in household**	1	Ref.			Ref.		
	More than 1	1.2	0.8–1.9	0.4	1.5	0.96–2.5	0.07
**Size of homestead land ***(in decimal*^a^*)*	Less than 5	Ref.			Ref.		
	5.1–20	1.3	0.97–1.9	0.07	1.2	0.8–1.7	0.3
	More than 20	1.3	0.9–2.0	0.2	1.1	0.7–1.8	0.6
**Size of agricultural land ***(in decimal*^a^*)*	None	Ref.			Ref.		
	0.1–100	0.7	0.5–0.97	0.03	0.6	0.4–0.9	0.009
	More than 100	0.6	0.4–0.9	0.01	0.5	0.3–0.8	0.003
**Education**	None	Ref.			Ref.		
	Partial/complete primary	1.6	1.1–2.5	0.02	1.4	0.9–2.1	0.09
	Secondary or more	2.2	1.4–3.4	< 0.001	2.1	1.3–3.2	0.002
**Number of children under 3 years**	No child	Ref.			Ref.		
	1 child	0.8	0.6–1.1	0.2	0.8	0.6–1.0	0.05
	2 children	0.7	0.4–1.3	0.2	0.7	0.4–1.4	0.3
**Empowerment**	None or very little	Ref.			Ref.		
	Some	1.1	0.8–1.5	0.6	1.4	0.96–1.9	0.09
	Greater	1.6	1.0–2.3	0.03	1.6	1.03–2.4	0.04

Total *n* = 1222, missing: 53, due to missing values in single variables

OR: odds ratio from mixed effects logistic regression model adjusting for clustering by settlement, CI: 95% confidence interval; Ref: reference category

^a^A decimal is a unit of area used in Bangladesh equal to 40.5 m^2^

## Discussion

The innovative food hygiene intervention was well accepted by the target population with high participation at all sessions. Women’s lack of mobility outside the home in this context could contribute to the slightly lower participation in group events than in the household visits [33]. One important factor in achieving a high overall participation could be the use of a wide variety of enjoyable activities during group events and household visits (e.g., role play, simulation games, demonstration of ideal food plate etc.). This finding resonated with earlier work showing that innovative and interactive methods are effective in ensuring a wide reach of hygiene interventions [14, 26, 35]. Also, the strong repeated interpersonal communication between trained food hygiene promoters and participants could have reinforced high acceptance. There is good evidence from different studies that as trusted members of the community, hygiene promoters can serve as catalysts in improving coverage and adoption of improved hygiene practices [36–41]. Finally, the use of rewards and social recognition (becoming an ‘ideal family’ or a ‘clean kitchen’ winner) could have encouraged participation in activities. Studies have shown that the use of positive recognition or reward is a strong motivation factor for participation in public health interventions and can stimulate desirable health behaviors [14, 26, 42]. In the present study, we observed that household participation at different sessions had a strong positive association with better adherence to food hygiene behaviors and consequently, becoming an ‘ideal family’ or a ‘clean kitchen’ winner. Families who participated in at least 7 out of 8 sessions showed more consistent practices of all four food hygiene behaviors until the end of the intervention compared to households with lower participation.

Although participation in intervention activities was high and all food hygiene practices increased compared with the pre-intervention period, our results suggest that adoption of different behaviors varied widely. For example, safe food storage practice increased between the pre-intervention and the first round of assessment but was not sustained and decreased in the two subsequent rounds. Cooking fresh or the reheating of stored foods and clean kitchen practices followed the same pattern. Overall, less than half of intervention households (~ 39%) maintained at least 5 of the 7 selected practices throughout the implementation period. This difference between participation in the intervention and adoption of actual behaviors is consistent with previous research on sanitation and hygiene and highlights that improved coverage or outreach in large-scale interventions does not necessarily equate to improved practice [41, 43].

According to various behavioral theories, a person’s initial decision to learn or adopt a new behavior is often influenced by their beliefs, values, attitudes, social norms, and networks, while retention of a behavior depends on their situational, material, social, and financial context [44, 45]. This phenomenon of “slippage in behavior” is well documented in other sanitation and hygiene studies in different contexts [46, 47] and is consistent with our finding that although some of the families had adopted food hygiene behaviors to improve their children’s health and nutrition, they were unable to maintain them consistently because of situational constraints, such as not having the time or energy between competing household tasks to cook or reheat food each time before feeding. Preference for a familiar activity [47, 48] due to convenience could also result in new behaviors being short-lived. This finding is supported by a similar study in Bangladesh, which showed that rural women preferred to keep food on the floor to make it easier to serve at meals, which are normally eaten sitting on the floor, even when they knew it was important to keep food in an elevated place to protect it from domestic animals and insects [49].

Changing the physical environment by disrupting old/familiar situational cues is therefore an important initial strategy in the development of a new behavior, which also helps people repeat the desired action many times in a stable context so that it can be performed automatically and more easily over time [13, 14, 50]. Based on this psychology of behavior change, at the beginning of our food hygiene intervention, we helped each participating household organize their kitchens or cooking areas by re-arranging food storage shelves, placing a mobile handwashing device with soap and water within easy reach and adding eye danglers as visual cues to interrupt unhealthy hygiene and kitchen habits. However, it was not easy for many study households to change their physical settings, especially in houses with no separate kitchen or cooking area, which could lead to instability in behavior-interrupting cues and thus limit the potential for adoption and sustained practices of new food hygiene behaviors [45].

Along with environmental modification, increasing access to supporting infrastructure and/or products remains critical to minimize physical barriers and thus encourage practice of certain food hygiene behaviors [51–53]. Handwashing with soap and cleaning of utensils are two such examples. Our analysis showed that handwashing before food preparation and cleaning of cooking and feeding utensils were practiced by less than half of our study population. This finding is supported by other studies in Bangladesh that observed that handwashing before food preparation is rarely done [11, 54–56]. In discussions with the FHPs and our study participants, suboptimal access to water and soap, especially near the kitchen area, was identified as an important physical barrier to handwashing before food preparation. There is also evidence from other studies that handwashing with soap before food preparation is strikingly higher if a functioning handwashing facility is conveniently located around the kitchen and eating area [51, 57, 58]. Similarly, access to water and soap can also facilitate keeping utensils clean [51, 52]. In our setting, proposed solutions by the team, e.g., to install tippy-taps [53] near the kitchen area, were not well accepted by study households. Producing a wet ground underneath the device was reported as a major inconvenience by study participants. Even though tippy-taps demonstrated success and promise for improving handwashing behavior in other projects in rural Bangladesh [59], this finding confirms that every setting is different and sustained use of an enabling behavior change technology largely depends on user’s needs, preferences, and motivation [53, 60]. Later, we pilot-tested a low-cost, locally available plastic sink with water storage tank and piped drainage system, installed in or nearby the kitchen with 10 intervention households and collected very positive qualitative feedback from the users. Future work will expand this to more households and conduct multiple rounds of testing and developing a locally acceptable, affordable and modifiable product (with different features and different cost levels) that can facilitate sustained handwashing and utensils cleaning practices in our population.

Apart from environmental or structural constraints, socio-economic factors also influence the likelihood of improved food hygiene practices. In the multivariable analysis, the practice of key food hygiene behaviors was strongly influenced by household wealth. This makes sense as cleaning of a separate and well-equipped kitchen area with water access and sink is likely easier compared to keeping the kitchen area of a one-room-house with no water access and mud floors in a clean state. This result is also in line with the fact that in Bangladesh poorer households are less likely to have a designated handwashing spot/station with water and soap than wealthier households [51, 61, 62].

Moreover, we found that the practice of key food hygiene behaviors increased with women’s education. This association remained strong even after adjusting for other woman- and household-level characteristics including wealth. The positive impact of women’s education on acceptance and utilization of nutrition, hygiene, and other health services has also been reported in many studies across multiple contexts [63–68]. In our study population, a higher level of education might help women understand the importance of safe food hygiene practices for their children’s health and thus to adopt promoted behaviors. Overall, we are still far from understanding all motivational drivers and barriers of food hygiene behavior change in our context and future qualitative studies might provide further necessary insights in this direction.

Nevertheless, the uptake of food hygiene behaviors among FAARM households was largely comparable to the Nepali study (39% in FAARM versus 42% in Nepal) [25] and the percentages of households with continuing practice of four food hygiene behaviors (i.e., handwashing, utensils cleaning, safe food storage and preparing fresh food or reheating stored food) were still considerably higher at the end of the intervention than pre-intervention. Future evaluations will show whether FAARM intervention households have been able to maintain these practices at current levels, thereby reducing food contamination and ultimately infections and diarrheal diseases in children.

### Strengths and limitations

A strength of this study was using direct observation of behaviors to assess the households’ food hygiene practices. The uptake of ‘ideal family’ and ‘clean kitchen’ behaviors were assessed over three rounds of observation during household visits. The results of these assessments should, however, be seen within their limitations. First, for practical reasons, the structured observation data was collected by the FHPs in the same households for which they were responsible for delivering services. Therefore, it is well possible that their knowledge on intervention objectives and familiarity with participating households could introduce observer bias and lead to an overestimation of actual practices [69–73]. Furthermore, participants may have felt inclined to improve their food hygiene-related behaviors in response to their awareness of being observed by their FHPs, resulting in social desirability bias [74–76]. To minimize these biases and ensure valid and reliable data collection across participating households, hygiene promoters received intensive training on the observation checklist, structured recording and coding of each behavior, and supportive supervision throughout the intervention. Third, the short duration of the structured observation, about 1 h per household, could limit the opportunity to observe multiple events related to different food hygiene behaviors [77, 78]. This time frame may also be too brief for participants to get used to the observer being present and return to normal behaviors [77–80]. Ideally, longer and more elaborate observations with independent observers should be performed. Nevertheless, the use of multiple rounds of structured observation as well as spot checks can provide some level of confidence in the objectivity of our assessment.

## Conclusion

We have shown that a proof-of-concept behavior change approach adapted from Nepal can be implemented on a large scale in another country and achieve satisfactory reach among participants. Overall, observed food hygiene practices improved compared to pre-intervention reported levels, with high participation identified as a key factor. However, different behaviors have been adopted to varying degrees, and lack of access to appropriate facilities and structures remains a major barrier to consistent practice. Future behavior change promotion should therefore consider combining this approach with appropriate enabling technologies, e.g., constructing a fixed designated low-cost hand-washing place near the kitchen or a good food storage cabinet using locally available materials, to improve food hygiene practices. This could be done through technical assistance or a cost-sharing approach to ensure community participation and ownership in developing solutions that work best for the households.

Although the intervention was implemented and evaluated in two rural sub-districts in northeastern Bangladesh, our findings are likely to be relevant to other settings with similar demographic characteristics across the country and beyond. We hope that sharing our findings can provide practical guidance to national and international organizations, stakeholders, and researchers in developing better interventions, facilitating context-specific adaptation, multi-sectoral implementation, and evaluation to improve child nutrition.

## Supplementary Information

**Additional file 1: Supplementary Table 1.** Data sources and collection timepoints.

**Additional file 2: Supplementary Table 2.** Participation in sessions of the food hygiene intervention.

**Additional file 3: Supplementary Figure 1.** Practice of ‘ideal family’ behaviors over time of intervention delivery. **Supplementary Figure 2.** Practice of ‘clean kitchen’ behaviors over time of intervention delivery.

**Additional file 4: Supplementary Table 3.** Crude associations of household characteristics with classification as ‘ideal family’ or ‘clean kitchen’ winner. **Supplementary Table 4.** Crude associations of women characteristics with classification as ‘ideal family’ or ‘clean kitchen’ winner.

**Additional file 5.** StaRI checklist.

## Data Availability

The datasets for the current study are available from the corresponding author on reasonable request.

## Abbreviations

FAARM: Food and Agricultural Approaches to Reducing Malnutrition
FGD: Focus group discussions
FHP: Food hygiene promoter
IDI: In-depth interviews
WASH: Water, sanitation and hygiene
