# Supplementary material for: Design, delivery, and determinants of uptake: findings from a food hygiene behavior change intervention in rural Bangladesh
Source: BMC Public Health. 2022 May 4;22:887. doi: 10.1186/s12889-022-13124-w (PMC9066747; doi:10.1186/s12889-022-13124-w)
Supplement: Supplementary file 1 — Additional file 1: Supplementary Table 1. Data sources and collection timepoints. [file 12889_2022_13124_MOESM1_ESM.pdf]

Supplemental Table 1. Data sources and collection timepoints

Data source	Dates	Data type	Data collected
FAARM baseline survey	March-May 2015	Survey	- Household wealth<br>- Household structure<br>- Number of rooms in the household<br>- Size of homestead and agricultural land<br>- Religion<br>- Women's education<br>- Women's empowerment
FAARM surveillance:<br>round 8 *(baseline for food hygiene practices and diverse diet)*	November-December 2016	Survey	- Four food hygiene behaviors<br>- Dietary diversity
FAARM surveillance:<br>round 10 and round 11 *(baseline for diverse garden practice)*	March-April 2017<br>May-June 2017	Survey	- Homestead garden diversity
FAARM surveillance:<br>round 11	May-June 2017	Survey	- Age of youngest child<br>- Total number of children under 3 years
Food hygiene field registers	July 2017-February 2018	Participation lists	- Attendance data
Household visit 2<br>Household visit 3<br>Household visit 4<br>*(Used for 'ideal family' and 'clean kitchen' competition)*	November 2017<br>December 2017<br>January 2018	Direct observation	- Current food hygiene practices
		Household spot checks	- Homestead garden diversity<br>- Cleanliness of kitchen and household environment<br>- Presence of a handwashing device<br>- Safe food storage facilities
		Survey<br>(24-hour recall)	- Dietary diversity
