# Supplementary material for: Design, delivery, and determinants of uptake: findings from a food hygiene behavior change intervention in rural Bangladesh
Source: BMC Public Health. 2022 May 4;22:887. doi: 10.1186/s12889-022-13124-w (PMC9066747; doi:10.1186/s12889-022-13124-w)
Supplement: Supplementary file 2 — Additional file 2: Supplementary Table 2. Participation in sessions of the food hygiene intervention. [file 12889_2022_13124_MOESM2_ESM.pdf]

Supplementary Table 2: Participation in events of the food hygiene intervention

Participation	freq.	%
Household visits		
visit 1	1228	96.3
visit 2 (observation round 1)	1176	92.2
visit 3 (observation round 2)	1143	89.7
visit 4 (observation round 3)	1085	85.1
Group events		
event 1	1093	85.7
event 2	1125	88.2
event 3	1026	80.5
event 4	1015	79.6

Total women: 1275
