# Supplementary material for: Design, delivery, and determinants of uptake: findings from a food hygiene behavior change intervention in rural Bangladesh
Source: BMC Public Health. 2022 May 4;22:887. doi: 10.1186/s12889-022-13124-w (PMC9066747; doi:10.1186/s12889-022-13124-w)
Supplement: Supplementary file 3 — Additional file 3: Supplementary Figure 1. Practice of ‘ideal family’ behaviors over time of intervention delivery. Supplementary Figure 2. Practice of ‘clean kitchen’ behaviors over time of intervention delivery. [file 12889_2022_13124_MOESM3_ESM.pdf]

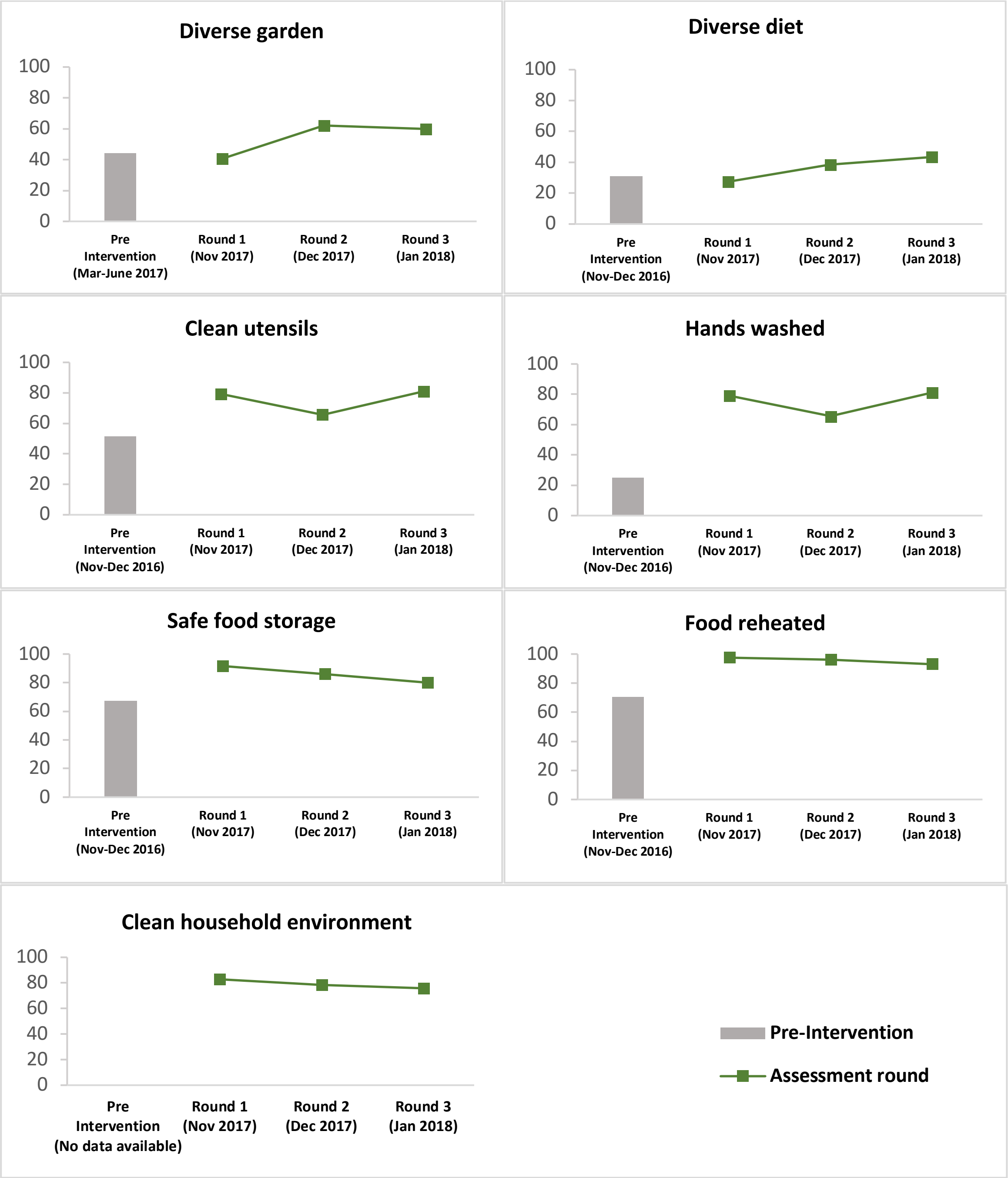

Supplementary Figure 1: Practice of 'ideal family' behaviors over time of intervention delivery.

Practice of 'ideal family' behaviors (in % of households) was measured over three rounds of assessment during the food hygiene intervention implementation through structured observations (green line). This graph only shows households that could be observed for 'ideal family' indicators during all three observation rounds (n=1022); households with less than 3 observation rounds were excluded (missing values: 253). Pre-intervention assessment data for four food hygiene behaviors and dietary diversity were collected during the routine surveillance in November–December 2016 and for diverse garden in March–June 2017 through interview questions in a sub-population of FAARM households (grey bar): diverse garden n=1301, diverse diet n=651, clean utensils n=653, hands washed n=448, safe food storage n=669, food reheated n=653.

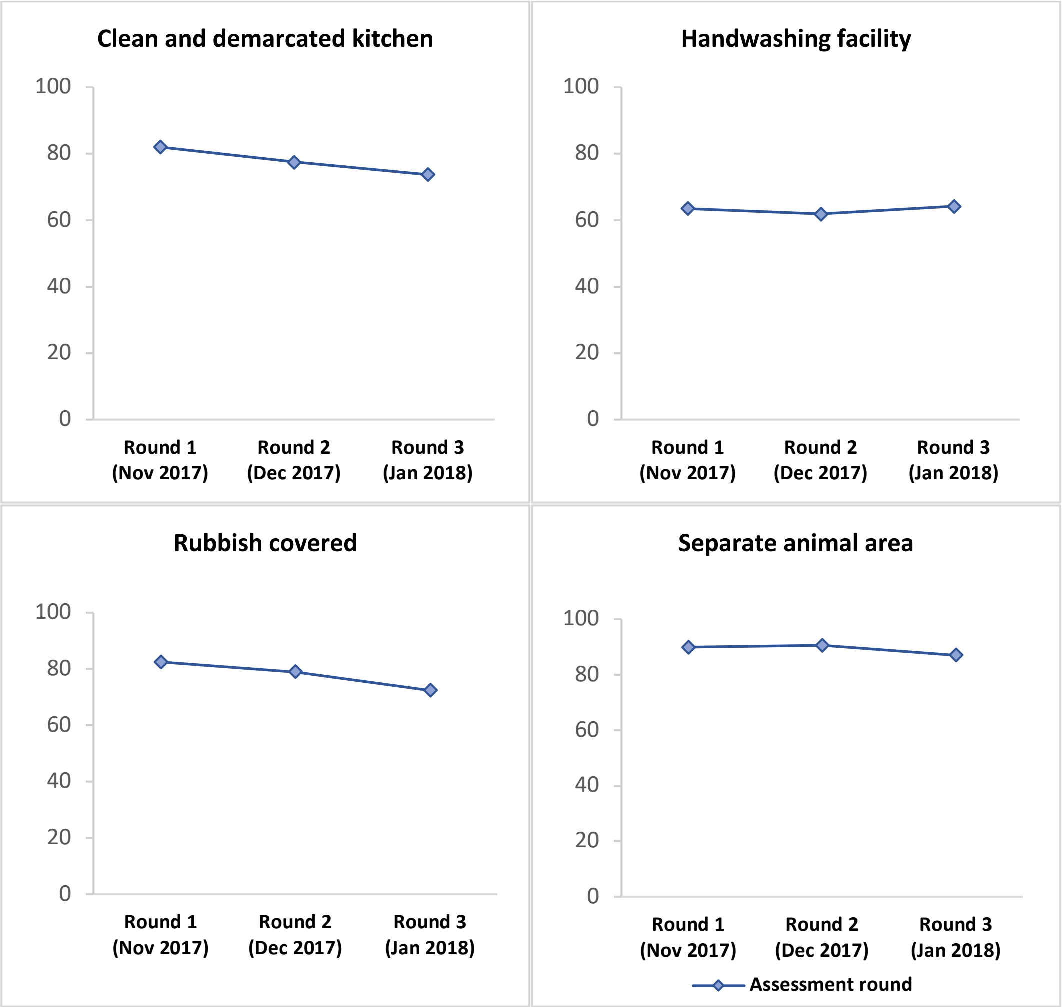

Supplementary Figure 2: Practice of 'clean kitchen' behaviors over time of intervention delivery. Practice of 'clean kitchen' behaviors (in % of households) was measured over three rounds of assessment during food hygiene intervention implementation through structured observations (blue line). This graph only shows households that could be observed for 'clean kitchen' indicators during all three observation rounds (n=1022); households with less than 3 observation rounds were excluded (missing values: 253).
