# Supplementary material for: Design, delivery, and determinants of uptake: findings from a food hygiene behavior change intervention in rural Bangladesh
Source: BMC Public Health. 2022 May 4;22:887. doi: 10.1186/s12889-022-13124-w (PMC9066747; doi:10.1186/s12889-022-13124-w)
Supplement: Supplementary file 4 — Additional file 4: Supplementary Table 3. Crude associations of household characteristics with classification as ‘ideal family’ or ‘clean kitchen’ winner. Supplementary Table 4. Crude associations of women characteristics with classification as ‘ideal family’ or ‘clean kitchen’ winner. [file 12889_2022_13124_MOESM4_ESM.pdf]

Supplementary Table 3: Crude associations of household characteristics with classification as 'ideal family' or 'clean kitchen' winner

		'Ideal family'				'Clean kitchen'			
**Household characteristics**		**freq. (%)**	**Crude OR**	**95% CI**	**p-value**	**freq. (%)**	**Crude OR**	**95% CI**	**p-value**
**Attendance**	Low	8 (7.7%)	Ref.			11 (10.6%)	Ref.		
	Medium	52 (26.7%)	4.0	1.8-8.9	<0.001	71 (36.4%)	6.5	3.0-13.8	<0.001
	High	436 (44.7%)	9.5	4.5-19.8	<0.001	567 (58.1%)	18.1	9.0-36.7	<0.001
**Wealth** *(in quintiles)*	Lowest	54 (19.1%)	*0.3*	0.2-0.5	<0.001	91 (32.2%)	*0.3*	0.2-0.5	<0.001
	Low	89 (34.9%)	0.8	0.6-1.2	0.56	124 (48.6%)	0.7	0.4-0.97	0.04
	Medium	100 (39.7%)	Ref.			139 (55.2%)	Ref.		
	High	130 (50.6%)	1.6	1.1-2.3	0.009	145 (56.4%)	1.1	0.7-1.6	0.74
	Highest	119 (54.8%)	1.9	1.3-2.8	0.001	147 (67.7%)	1.8	1.2-2.7	0.008
**Religion**	Muslim	310 (34.5%)	Ref.			415 (46.2%)	Ref.		
	Hindu	185 (49.6%)	1.9	1.4-2.5	<0.001	234 (62.7%)	2.6	1.8-3.8	<0.001
**Household members**	Up to 5	161 (36.1%)	Ref.			227 (50.9%)	Ref.		
	5-10	214 (36.6%)	1.0	0.7-1.3	0.84	291 (49.7%)	0.9	0.7-1.2	0.46
	More than 10	117 (50.2%)	1.8	1.3-2.5	<0.001	128 (54.9%)	1.3	0.9-1.9	0.1
**Number of rooms in household**	1	57 (24.1%)	Ref.			87 (36.7%)	Ref.		
	More than 1	420 (42.1%)	2.3	1.6-3.2	<0.001	540 (54.2%)	2.3	1.7-3.1	<0.001
**Size of homestead land** *(in decimal*)*	Up to 5	124 (29.6%)	Ref.			183 (43.7%)	Ref.		
	5.1 - 20	230 (42.4%)	1.7	1.3-2.3	<0.001	296 (54.5%)	1.6	1.2-2.1	0.002
	More than 20	123 (45.2%)	2.0	1.5-2.9	<0.001	148 (54.4%)	1.7	1.2-2.5	0.002
**Size of agricultural land** *(in decimal*)*	None	173 (33.3%)	Ref.			251 (48.3%)	Ref.		
	0.1-100	103 (36.3%)	1.1	0.8-1.5	0.5	136 (47.9%)	1.0	0.8-1.4	0.75
	More than 100	201 (46.7%)	1.8	1.4-2.4	0.001	240 (55.8%)	1.6	1.2-2.2	0.001

*Total n= 1275; for some variables total n is smaller due to additional missing data: wealth and number of household members: n=1264, religion: n=1271, number of rooms in the household, size of homestead land and size of agricultural land: n=1234; OR: odds ratio from mixed effects logistic regression model adjusting for clustering by settlement, CI: 95% confidence interval; Ref: reference category. *A decimal is a unit of area used in Bangladesh equal to 40.5 m^2.*

Supplementary Table 4: Crude associations of women characteristics with classification as 'ideal family' or 'clean kitchen' winner

		'Ideal family'				'Clean kitchen'			
**Women characteristics**		**freq. (%)**	**Crude OR**	**95% CI**	**p-value**	**freq. (%)**	**Crude OR**	**95% CI**	**p-value**
**Education**	None	40 (20.2%)	Ref.			64 (32.3%)	Ref.		
	Partially/complete primary	201 (35.8%)	2.2	1.5-3.3	<0.001	269 (47.9%)	2.0	1.4-2.9	<0.001
	Partially secondary or more	254 (49.7%)	4.0	2.7-6.0	<0.001	316 (61.8%)	3.9	2.7-5.8	<0.001
**Number of children under three years**	No child	279 (40.7%)	Ref.			367 (53.6%)	Ref.		
	1 child	198 (37.9%)	0.9	0.7-1.1	0.31	253 (48.5%)	0.9	0.7-1.1	0.20
	2 children	19 (27.9%)	0.6	0.3-0.99	0.05	29 (42.6%)	0.6	0.4-1.1	0.12
**Age of youngest child** *(under three years)*	0-5 months	33 (39.8%)	Ref.			45 (54.2%)	Ref.		
	6-11 months	37 (29.6%)	0.6	0.3-1.2	0.13	52 (41.6%)	0.6	0.3-1.1	0.08
	12-17 months	29 (33.7%)	0.7	0.4-1.4	0.35	33 (38.4%)	0.5	0.3-1.1	0.08
	18-23 months	34 (32.7%)	0.8	0.4-1.4	0.41	44 (42.3%)	0.7	0.4-1.3	0.28
	24-29 months	35 (40.2%)	1.0	0.5-2.0	0.90	49 (56.3%)	1.3	0.6-2.5	0.48
	30-36 months	49 (46.7%)	1.3	0.7-2.4	0.40	59 (56.2%)	1.2	0.6-2.4	0.52
**Empowerment**	None or very little	106 (38.0%)	Ref.			133 (47.7%)	Ref.		
	Some	248 (36.9%)	1.0	0.7-1.3	0.76	343 (51.0%)	1.1	0.8-1.5	0.60
	Greater	126 (43.4%)	1.3	0.9-1.8	0.14	154 (53.1%)	1.3	0.9-1.9	0.11

Total n= 1275; for some variables total n is smaller due to additional missing data: women's education: n=1271, empowerment: n= 1241; OR: odds ratio from mixed effects logistic regression model adjusting for clustering by settlement, CI: 95% confidence interval; Ref: reference category.
